# Detection of NUCB2/nesfatin-1 in cerebrospinal fluid of multiple sclerosis patients

**DOI:** 10.18632/aging.202287

**Published:** 2020-12-12

**Authors:** Masaru Shimizu, Taizo Manome, Masumi Kumami, Kouzou Matsumura, Kazuaki Kanai, Kenju Shimomura, Yuko Maejima

**Affiliations:** 1 Department of Bioregulation and Pharmacological Medicine, Fukushima Medical University School of Medicine, Fukushima, Japan; 2 Department of Neurology, Fukushima Medical University School of Medicine, Fukushima, Japan; 3 Department of Neurology, Matsumura General Hospital, Fukushima, Japan

**Keywords:** NUCB2/nesfatin-1, multiple sclerosis, inflammation

## Abstract

NUCB2/nesfatin-1 was originally discovered as an anorexigenic peptide. However, recent studies revealed various additional functions including the regulation of inflammation. However, there are no studies that investigated the involvement of NUCB2/nesfatin-1 in neuroinflammatory diseases. Here, we aimed to investigate the involvement of NUCB2/nesfatin-1 in a representative neuroinflammatory disease, multiple sclerosis (MS).

Cerebrospinal fluids (CSF) were collected from 24 MS patients and 10 control subjects and NUCB2/nesfatin-1, proinflammatory cytokines (TNF-α, IL-1β) and anti-inflammatory cytokines (IL-10, TGF-β) levels were measured by using ELISA assay. Also the expression of NUCB2/nesfatin-1 in the CSF of MS patient was investigated by western blot analysis.

Expression of NUCB2/nesfatin-1 was confirmed in the CSF of the MS patient by western blot analysis. NUCB2/nesfatin-1 levels were significantly higher in the CSF of the MS patients. Among the measured cytokines, only IL-1β was lower in the CSF of the MS patients.

We report for the first time increased NUCB2/nesfatin-1 levels in the CSF of MS patients.

## INTRODUCTION

Multiple sclerosis (MS) is an inflammatory disease affecting the central nervous system (CNS) of more than 2 million people worldwide [1]. Its symptoms vary from physical to mental such as visual problems, depression, anxiety and fatigue [2].

Although detailed pathological mechanisms still remain unclear, the key factor is considered to be dysfunction of the immune system associated with CNS inflammation and demyelination which ultimately leads to axonal damage and destruction of the neural network [3]. Recent studies have revealed the possible interference of inflammation related molecules and cytokines such as interleukin (IL)-1β, IL-6, IL-8, and tumor necrosis factor (TNF) in the induction of demyelination [4–6].

The major challenge of diagnosing MS is that clinical symptoms and results of imaging examinations vary substantially between patients [7].

As mentioned above, because neural inflammation is the key factor for developing MS, proinflammation and anti-inflammation cytokines in the cerebrospinal fluid (CSF) may be associated with the disease and may be useful as biomarkers to diagnose MS. In fact, recent studies reported the elevation of proinflammatory molecules in MS patients [4–6, 8].

On the other hand, nesfatin-1 is an 82-amino-acid peptide derived from NEFA/nucleobindin2 (NUCB2) which is expressed in both the peripheral and CNS [9]. NUCB2/nesfatin-1 was originally identified as an endogenous anorexigenic peptide but recent studies have indicated various additional functions aside from feeding regulation [9–16]. Among such newly discovered functions of NUCB2/nesfatin-1, some studies indicated possible involvement in anti-inflammation mechanism [17–19].

In this study, we collected the CSF from MS patients and examined the level of NUCB2/nesfatin-1 together with proinflammatory and anti-inflammatory cytokines. Our present results may indicate the potentials of NUCB2/nesfatin-1 as a biomarker for diagnosing MS. Also, the results of inflammation related cytokine levels provide insight into the pathological mechanism and the involvement of inflammation in developing MS.

## RESULTS

### NUCB2/nesfatin-1 levels of CSF from MS patients

The mean ages were 43.2 + 1.8 for the MS patients and 36.5 + 5.1 for the control subjects.

**Figure 1:**
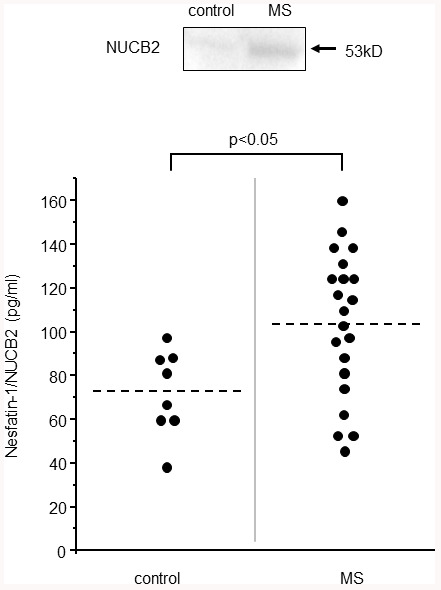
**NUCB2/nesfatin-1 in the CSF of the MS patients.** The expression of NUCB2/nesfatin-1 in the CSF of MS patient confirmed by western blot analysis (above). The NUCB2/nesfatin-1 levels in the control (n=10) and MS patients (n=24).

As shown in Figure 1 (above), expression of NUCB2/nesfatin-1 was confirmed in western blot analysis. The levels of NUCB2/nesfatin-1 were 38.2 and 138.3 pg/ml in the control subjects and MS patients, respectively.

Analysis of the CSF showed significantly higher levels of NUCB2/nesfatin-1 in the MS patients (Figure 1; p=0.02) than in the control subjects. Serum and CSF levels of NUCB2/nesfatin-1 had no significant correlations (r = -0.6, P = 0.09).

**Figure 2:**
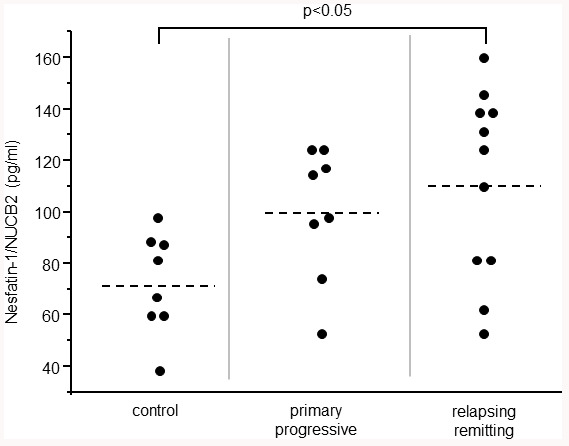
**NUCB2/nesfatin-1 in the CSF of the control subjects, patients with primary progressive MS and relapsing remitting MS.**

When comparing the levels of NUCB2/nesfatin-1 in control subjects, patients with primary progressive MS and relapsing remitting MS, average NUCB2/nesfatin-1 level was significantly higher in CSF of relapsing remitting MS patients (Figure 2). There were no differences in levels of NUCB2/nesfatin-1 among each phenotypes of MS (Clinically isolated syndrome, relapsing-remitting MS, Primary progressive MS, Secondary progressive MS).

There were 2 subjects under steroid pulse treatment when CSFs were collected (which were excluded from results shown in Figures 1, 2). These two subjects showed low levels of NUCB2/nesfatin-1 that were undetectable by the assay kit we have used in this study.

### Levels of proinflammatory cytokines in CSF of MS patients

**Figure 3:**
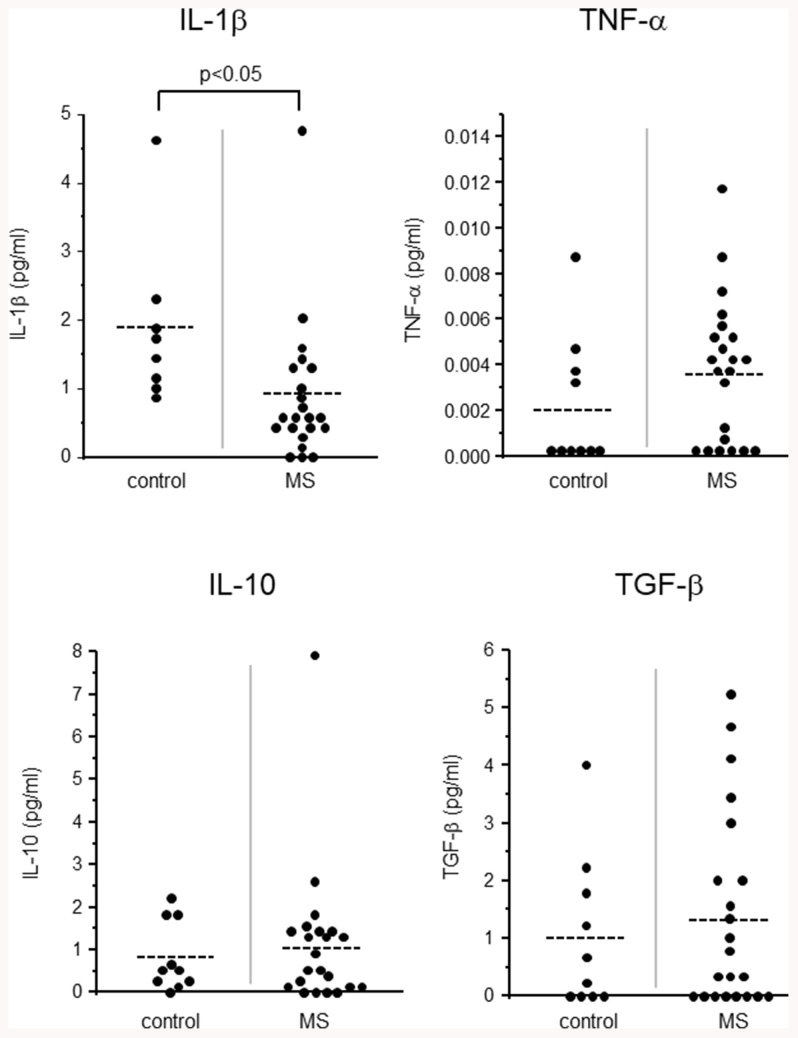
**Levels of proinflammatory cytokines (IL-1β, TNF-α; above) and anti-inflammatory cytokines (IL-10, TGF-β; below).**

We have measured the levels of IL-1β and TNFα as proinflammatory cytokines in the CSF. TNFα levels were not significantly different between the MS patients and control subjects (Figure 3 above right). However, to our surprise, IL-1β level of the CSF from the MS patients was significantly lower than that of the control subjects (Figure 3 above left).

### Levels of anti-inflammatory cytokines in CSF of MS patients

We also measured the levels of IL-10 and TGF-β as anti-inflammatory cytokines in the CSF. Both IL-10 and TGF-β had no significant difference between the MS patients and control subjects (Figure. 3 below).

## DISCUSSION

In this study, we have shown increased NUCB2/nesfatin-1 level in the CSF of MS patients. Although the number of subjects is relatively small, to the best of our knowledge, this is the first study to evaluate NUCB2/nesfatin-1 levels in the CSF of human patients with neuroinflammatory disease.

It is considered that some form of immunological reaction may initiate neuroinflammation that ultimately leads to demyelination and neurodegeneration in the CNS [2, 20, 21]. Recently, many studies have been performed to evaluate the interference of inflammatory factors in the CSF of MS patients [4–6]. In these studies, proinflammatory conditions of the CSF were shown to be a critical factor that induces and maintains the disease. Proinflammatory cytokines such as IL-6 and IL-8 are reported to be related to long-term disease activity and progression of symptoms of MS [5]. It is thus obvious that existence of inflammation and inflammatory cytokines in the CSF are the key factors to understand the mechanism of developing MS.

NUCB2/nesfatin-1, which was originally discovered as an anorexigenic peptide is now considered to have various functions in both the CNS and peripheral tissues [9–16]. These new functions include regulations of blood pressure, glucose homeostasis and even cardiac performance [11–16]. In addition, regulation of inflammation by NUCB2/nesfatin-1 has been reported. Naseroleslami et al. reported that in cardiomyocytes NUCB2/nesfatin-1 attenuates myocardial infarction by reducing proinflammatory cytokines [17]. Also, Jiang et al. showed that NUCB2/nesfatin-1 can ameliorate osteoarthritis by suppressing inflammation [19]. These reports indicate the ability of NUCB2/nesfatin-1 to suppress inflammation. In the present study, we found that NUCB2/nesfatin-1 level in the CSF is increased in MS patients. The results showing NUCB2/nesfatin-1 level in the CSF of patients under steroid pulse treatment was undetectably low in assay kit we have used in this study, further support the involvement of NUCB2/nesfatin-1 on inflammation. Because exogenously applied NUCB2/nesfatin-1 is reported to suppress inflammation of post traumatic brain injury [22], increase of NUCB2/nesfatin-1 which was found in this study is likely to reflect the anti-inflammatory response rather than result of neuroinflammation itself. Further study is required to elucidate the detailed mechanism for the elevation of NUCB2/nesfatin-1 level in the CSF of MS patients.

To our surprise, a proinflammatory cytokine, IL-1β, was found to be significantly reduced in the CSF of MS patients. Because of ongoing inflammation in MS patients, the reduction of proinflammatory cytokine IL-1β was unexpected. In the past, Rossi et al. reported the detection of IL-1β in the CSF of MS patients only in the remission phase [4]. The present study analyzed CSF samples from both primary and remitted MS patients but IL-1β level was clearly and significantly reduced in both patients. This may be explained by the elevation of NUCB2/nesfatin-1 level in the CSF. Jiang et al. reported that NUCB2/nesfatin-1 can suppress IL-1β induced inflammation [19]. Therefore NUCB2/nesfatin-1 and IL-1β levels may have some functional connection in the CSF of MS patients. Further study is required.

Up to present, diagnosis of MS is based on symptoms and brain images obtained from MRI. In addition, oligoclonal bands of IgG on electrophoresis of the CSF are an effective marker for its diagnosis [7]. There are reports of other possible CSF biomarkers to diagnose MS but discovery of a promising new biomarker is expected. As a limitation of this study, the number of participants of this study is small and further large scale investigation is required. However, our present data show for the first time that level of NUCB2/nesfatin-1 in CSF may have a potential to be an effective biomarker for diagnosis of MS.

## MATERIALS AND METHODS

### MS patient samples

CSF samples were collected from 24 MS patients (7 male and 17 female) admitted to the Department of Neurology in Matsumura General Hospital. As control, 10 CSF samples were collected from patients affected by non-inflammatory neurological diseases (patients with strong headache or conversion disorder: 4 males and 6 females). The study was approved by the ethics committee of Fukushima Medical University, School of Medicine and Matsumura General Hospital, according to the Declaration of Helsinki. All patients were provided written informed consent to this study. MS diagnosis was made according to 2017 McDonald Criteria for Diagnosis of Multiple Sclerosis, which is based on the combinations of clinical presentation and additional data (Table 1) [7].

**Table 1:** Diagnostic criteria for MS.

**Clinical presentation**	**Additional data needed for diagnosis**
≥2 clinical attacks and objective evidence of 1 lesion	Disseminated in space (DIS): an additional attack implicating a different CNS site OR by MRI (≥ new lesions on follow-up imaging both gadolinium-enhancing and non-enhancing lesions on single MRI.)
1 clinical attack and objective clinical evidence of ≥ 2 lesions	Disseminated in time (DIT): an additional clinical attack implicating a different CNS site OR by MRI (≥1symptomatic or asymptomatic lesion in ≥2 areas including cortical/juxtacortical, periventricular, infratentorial, or spinal.) **OR** CSF-specific oligoclonal bands
1 clinical attack and objective evidence of 1 lesion	DIS:an additional clinical attack implicating a different CNS site OR by MRI (≥ new lesions on follow-up imaging both gadolinium-enhancing and non-enhancing lesions on single MRI.) **OR** DIT:an additional clinical attack OR by MRI (≥1symptomatic or asymptomatic lesion in ≥2areas including cortical/juxtacortical, periventricular, infratentorial, or spinal.) **OR** CSF-specific oligoclonal bands

### Western blot analysis

Twenty μl of CSF samples dissolved in 2 X SDS sample buffer was loaded onto a 10% polyacrylamide gel (156103, Bio-Rad, CA, USA). The samples were separated at 20 mA. Proteins were transferred from the gel onto a PVDF membrane at 118 mA for 60 min with transfer buffer. The membrane was washed several times with PBS containing 0.05% of Tween-20 (PBST) and blocked with 5% skim milk in PBST for 60 min at room temperature. Then the membrane was incubated with a rabbit polyclonal NUCB2 antibody (1:1000, N9414, Sigma-Aldrich, MO, USA) for overnight at 4° C. The antibody was diluted in blocking solution. The membrane was washed with PBST and incubated with HRP-conjugated secondary antibodies against rabbit IgG (1:1000, Vector Laboratories, CA, USA) for 60 min at room temperature. Then the membrane was washed several times and peroxidase activity was detected.

### ELISA assay

Levels of NUCB2/nesfatin-1 (Abcam, Cambridge, UK), IL-1β, TNFα, IL-10 and TGF-β (Biolegend, San Diego, CA, USA) in CSF and serum were measured by commercially available ELISA assay kit.

### Statistical analysis

All data are presented as mean. Statistical analysis was made by student’s *t*-test and one-way ANOVA followed by Tukey test for Figure 2. P<0.05 was considered significant.
